# Shedding light on neurofilament involvement in cognitive decline in obstructive sleep apnea and its possible role as a biomarker

**DOI:** 10.3389/fpsyt.2023.1289367

**Published:** 2023-11-30

**Authors:** Julia Jaromirska, Piotr Kaczmarski, Dominik Strzelecki, Marcin Sochal, Piotr Białasiewicz, Agata Gabryelska

**Affiliations:** ^1^Department of Sleep Medicine and Metabolic Disorders, Medical University of Lodz, Lodz, Poland; ^2^Department of Affective and Psychotic Disorders, Medical University of Lodz, Lodz, Poland

**Keywords:** obstructive sleep apnea, chronic intermittent hypoxia, neurofilaments, cognitive impairment, biomarkers, neurodegeneration

## Abstract

Obstructive sleep apnea is one of the most common sleep disorders with a high estimated global prevalence and a large number of associated comorbidities in general as well as specific neuropsychiatric complications such as cognitive impairment. The complex pathogenesis and effects of the disorder including chronic intermittent hypoxia and sleep fragmentation may lead to enhanced neuronal damage, thereby contributing to neuropsychiatric pathologies. Obstructive sleep apnea has been described as an independent risk factor for several neurodegenerative diseases, including Alzheimer's disease and all-cause dementia. The influence of obstructive sleep apnea on cognitive deficits is still a topic of recent debate, and several mechanisms, including neurodegeneration and depression-related cognitive dysfunction, underlying this correlation are taken into consideration. The differentiation between both pathomechanisms of cognitive impairment in obstructive sleep apnea is a complex clinical issue, requiring the use of multiple and costly diagnostic methods. The studies conducted on neuroprotection biomarkers, such as brain-derived neurotrophic factors and neurofilaments, are recently gaining ground in the topic of cognition assessment in obstructive sleep apnea patients. Neurofilaments as neuron-specific cytoskeletal proteins could be useful non-invasive indicators of brain conditions and neurodegeneration, which already are observed in many neurological diseases leading to cognitive deficits. Additionally, neurofilaments play an important role as a biomarker in other sleep disorders such as insomnia. Thus, this review summarizes the current knowledge on the involvement of neurofilaments in cognitive decline and neurodegeneration in obstructive sleep apnea patients as well as discusses its possible role as a biomarker of these changes.

## 1 Introduction

Obstructive sleep apnea (OSA) refers to a common sleep-related breathing disorder characterized by recurrent episodes of apneas and hypopneas, resulting from repetitive upper-airway collapse (1). The estimated global prevalence of OSA ranges from 9 to 38%, and most cases remain undiagnosed due to non-specific manifestations, such as snoring, drowsiness, or excessive daytime sleepiness (2). Chronic intermittent hypoxia (CIH) and sleep fragmentation, as the main consequences of OSA, lead to enhanced neuronal damage and a decrease in synaptic plasticity, thereby contributing to neurological pathologies (3). The untreated disorder is an independent risk factor for (4, 5) any neurocognitive disorder, Alzheimer's disease, and Parkinson's disease (6). Since not every patient experiences these disorders, the potential influence of compensatory mechanisms among OSA patients was reported (7). Continuous positive airway pressure (CPAP) as the first-line treatment for OSA patients has been considered an efficacious method for preserving cognitive function in some articles (8, 9); however, it should be noted that not all patients can benefit from the proposed change and the available data are not conclusive enough to support its effectiveness (10).

**Figure 1:**
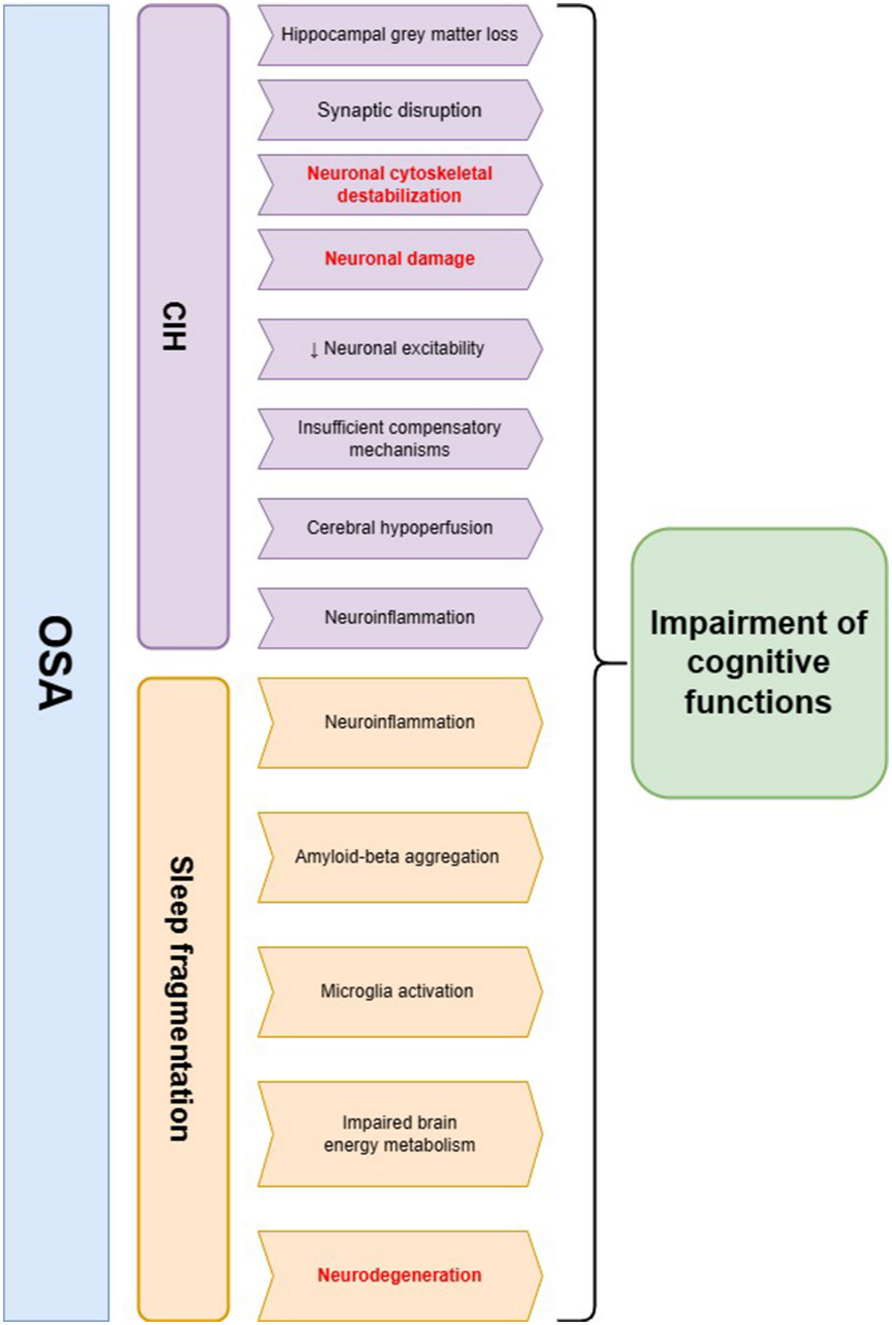
The summary of pathophysiological pathways leading to cognitive impairment in obstructive sleep apnea (OSA) patients. Two main hallmarks of OSA—chronic intermittent hypoxia (CIH) and sleep fragmentation—may influence cognitive decline through several mechanisms. The main hypotheses regarding the involvement of CIH in this process include neuronal cytoskeletal destabilization, synaptic disruption, neuroinflammation, and cerebral hypoperfusion, which possibly lead to neuronal injury and a decline in cognitive functions. Sleep fragmentation in OSA patients may evoke neurodegenerative changes including amyloid-beta aggregation as well as microglia activation. Additionally, it may lead to impaired brain energy metabolism and neuroinflammation, which are the possible risk factors of cognitive decline. The changes in neurofilament levels may be a possible biomarker of pathomechanisms underlined in red color.

Cognitive functioning refers to a collection of mental processes (e.g., perception, attention, memory, reasoning, and language skills) that are essential in comprehending the surrounding environment and acquiring knowledge. Cognitive performance declines with normal aging due to the accumulation of damage—neuronal structure alterations, synapse loss, and neuronal network dysfunction; however, it can be accelerated by some disorders (11). As a hallmark of OSA, CIH triggers cerebral hypoperfusion, endothelial dysfunction, and neuroinflammation, leading to blood-brain barrier dysfunction and cerebral small vessel disease; sleep fragmentation enhances the negative influence by increasing the production of oxygen species, coagulation, and sympathetic activity (12). Patients with OSA are more likely to exhibit deficits in attention and vigilance, long-term memory impairment, visuospatial dysgnosia, and executive dysfunction (13). The most prominent brain damage occurs in the prefrontal cortex, which is in charge of executive functions, and the greatest loss of gray matter localizes in the right basolateral amygdala/hippocampus (14, 15) (as shown in Figure 1).

Opinions are divided on whether all cognitive deficits in OSA are associated with neuronal damage, pointing at pseudodementia as a culprit of abnormalities (16). Patients with OSA due to general deterioration in life quality are more likely to suffer from depressive symptoms which affect the cognitive performance. The differentiation between the early signs of neurodegeneration and depression-related cognitive dysfunction can be challenging and expensive to identify. The clinical symptoms associated with this condition are often indistinguishable, making the diagnosis a time-consuming and expensive process that may involve magnetic resonance imaging. Currently, the optimal diagnostic method has not yet been developed; however, detecting peripheral blood or cerebrospinal fluid biomarkers associated with the neuronal condition can be promising as a non-invasive and cost-effective solution for identifying cognitive decline even in the earliest stages, tracking deficits' progression, and predicting reversibility chances. Biomarkers of neuroprotection, for e.g., brain-derived neurotrophic factor (BDNF), insulin-like growth factor 1, and neurofilaments (NFs), as well as Alzheimer's disease-related biomarkers, for e.g., amyloid-β 42, phosphorylated tau, and total tau, are gaining ground (17–24). The determination of especially NFs (possible from CSF and/or plasma/serum), due to their specificity and ease of measurement, along with cognitive testing appears to be prospective in cognition assessment.

We conducted a review of current literature regarding biomarkers of neuroprotection and neurodegeneration, specifically on neurofilaments. Additionally, we examined the involvement of neurofilaments in sleep disorders with a particular emphasis on OSA and cognitive disorders associated with it. We searched the following databases using the following search queries: PubMed (search queries: “neurofilaments,” “sleep disorders and neurofilaments,” “OSA and neurofilaments,” “BDNF and OSA,” “neurodegeneration and OSA,” and “neurodegeneration and neurofilaments”); Google Scholar (search queries: “OSA and neurofilaments,” “sleep disorders and neurofilaments,” “insomnia and neurofilaments,” “neurofilaments and neurodegeneration,” “neurofilaments and OSA and cognition and biomarker,” “OSA and BDNF,” and “neurofilaments and sleep architecture”).

In this study, we aim to summarize the recent data regarding the involvement of NFs in the neurodegenerative processes in OSA. Furthermore, it is important to review the existing literature describing the usefulness of NF plasma concentration as a possible biomarker of cognitive decline in OSA patients.

## 2 Neurofilament biology

The neurofilaments in the mature nervous system belong to a type IV intermediate filament family composed of quadruplet proteins—heavy, medium, light chain neurofilaments (abbreviated as NfH, NfM, and NfL, and encoded by NEFH, NEFM, and NEFL genes), and internexin (25). They are neuron-specific scaffolding subunits highly expressed in axons, providing structural stability vital to the outgrowth of axons (26). In addition, the NF structure consists of a highly conserved central domain composed of 310 amino acids specific to all intermediate filaments, providing a coiled-coil structure, a C-terminus with multiple lysine-serine-proline (KSP) motifs, and an N-terminus (27). The NF synthesis occurs constantly in the neurons' soma and the subunits combine to form 10-nm-diameter cytoplasmatic polymers, which are transported along axons by slow axonal transport (28). After reaching their destination, NFs undergo phosphorylation and glycosylation. The most extensive modifications occur in KSP repeats located in the tail. Both transport defects and phosphorylation disturbances were reported to cause neurodegeneration (29).

Impaired glucose metabolism was reported to trigger hyperphosphorylation of NFs, although there was no significant difference in overall NF levels; the change was accompanied by decreased protein O-GlcNAcylation in the cerebrum and tau hyperphosphorylation (30). The reciprocal relationship between O-GlcNAcylation and the level of NF phosphorylation at the head and tail domains containing KSP repeat motifs indicated the influence of post-transcriptional modification on the protein structure, resulting in shorter NFs with less assemblage, accumulating in somas rather than axons. In addition, improving O-GlcNAcylation as a target of treatment may result in fewer hyperphosphorylated NFs (31). Furthermore, phosphatase and tensin homolog/phosphorylated protein kinase B pathway dysregulation with cyclin-dependent kinase 5 and extracellular signal-regulated kinase involvement were connected with overall tau and NF hyperphosphorylation (32). Notably, the activation of extracellular signal-regulated kinase contributes to oxidative stress-triggered NF damage, which is found to be increased among OSA patients (33–35). Increased levels of non-phosphorylated NFs can alter homeostasis and contribute to neuronal degeneration; however, the exact etiology remains to be elucidated (36). The dysregulation of heat shock protein Hsp27 and alphaB-crystallin were reported to possibly take part in NFs' structure disturbances via kinase and phosphatase activity imbalance, leading to enhanced aggregation (37).

Among other cytoskeletal proteins (such as microtubules, actin filaments, and associated proteins), NFs are only specific to neurons, thereby making them useful indicators for non-invasive assessment of the brain condition by analogy with troponins in heart damage. NfL is the most abundant and soluble subunit of the neurofilament family, and therefore, it is the most studied. Neuro-axonal damage causes a release of NFs into the cerebrospinal fluid (CSF) and subsequently into the bloodstream. This may occur either passively or using exosomes (38). Compared to other brain injury biomarkers, such as BDNF or tau protein, NFs are not produced in the periphery, which can easily reflect the protein brain levels without errors (39). As we age, it is inevitable that thousands of neurons degenerate and are lost every day under normal circumstances Therefore, CSF/serum NFs maintain a relatively low constant level; however, pathologic conditions resulting in enhanced neuronal death increase the biomarker levels. NfL elevated levels have been observed in various neurological conditions, including Alzheimer's disease, mild cognitive impairment, frontotemporal dementia, amyotrophic lateral sclerosis, and multiple sclerosis (40–44).

## 3 Neurofilaments in sleep

Healthy sleep possesses neuroprotective effects in healthy individuals by participating in proper waste clearance, regaining neuronal sensitivity, and preventing reactive oxygen species overproduction (45). There is limited data available on the impact of sleep quality and related sleep disturbances on NFs. However, sleep disorders were reported to increase the risk of Alzheimer's disease, vascular dementia, and all-cause dementia (46). Sleep disturbances can be one of the first symptoms of ongoing brain damage, without other diagnostics (47).

### 3.1 Neurofilament and sleep loss/insomnia

Sleepiness can be associated with increased NfL levels, indicating a self-perpetuating cycle. The dependency may arise from the initial neuronal disruption that occurred independently from a sleep disorder and caused sleepiness through related neural network damage. Another explanation can start from exogenous stimuli, such as sleep depletion, which in consequence injure neurons and induce a release of NFs from cells (48). In general, sleep loss results in a depletion of global cognitive capacities, particularly reflected in attention and psychomotor vigilance impairment (49). In one study, one-night sleep loss among healthy subjects did not alter the levels of NfL, glial fibrillary acidic protein (GFAP), β-amyloid (Aβ) 40, and Aβ42; however, tau protein blood level was found to be increased (50). Another study indicated that serum levels of NfL in healthy subjects were significantly higher following one night of total sleep deprivation in a laboratory setting. Although the cognitive assessment did not statistically differ depending on the weight, obese subjects had increased pTau-181 levels, a biomarker of neurodegeneration (51). Apart from OSA participants described in this report, patients with chronic insomnia disorder exhibited higher serum levels of NfL, NfH, and NSE, which decreased after 6 months of treatment, indicating a possibility of reversal of the changes; the S100B protein level remained increased without significant alteration. The free therapy group of patients had lower cognitive performance scores measured by the Montreal Cognitive Assessment (MoCA) test compared to controls and those who underwent therapy (52). A different study found that levels of serum NfL and leukocyte telomere shortening (used as an aging marker) had been associated with female chronic insomnia patients' total sleep time, sleep efficiency, and sleep quality, measured by the Pittsburgh Sleep Quality Index (53). Contrarily, NF levels were not informative in narcolepsy type I, and sleep quality assessment, which was measured by the Medical Outcomes Study Sleep Scale, was altered in patients with Alzheimer's disease, suggesting an important role of sleep loss in NF alternations (54, 55).

### 3.2 Neurofilament and sleep architecture disruption

**Figure 2:**
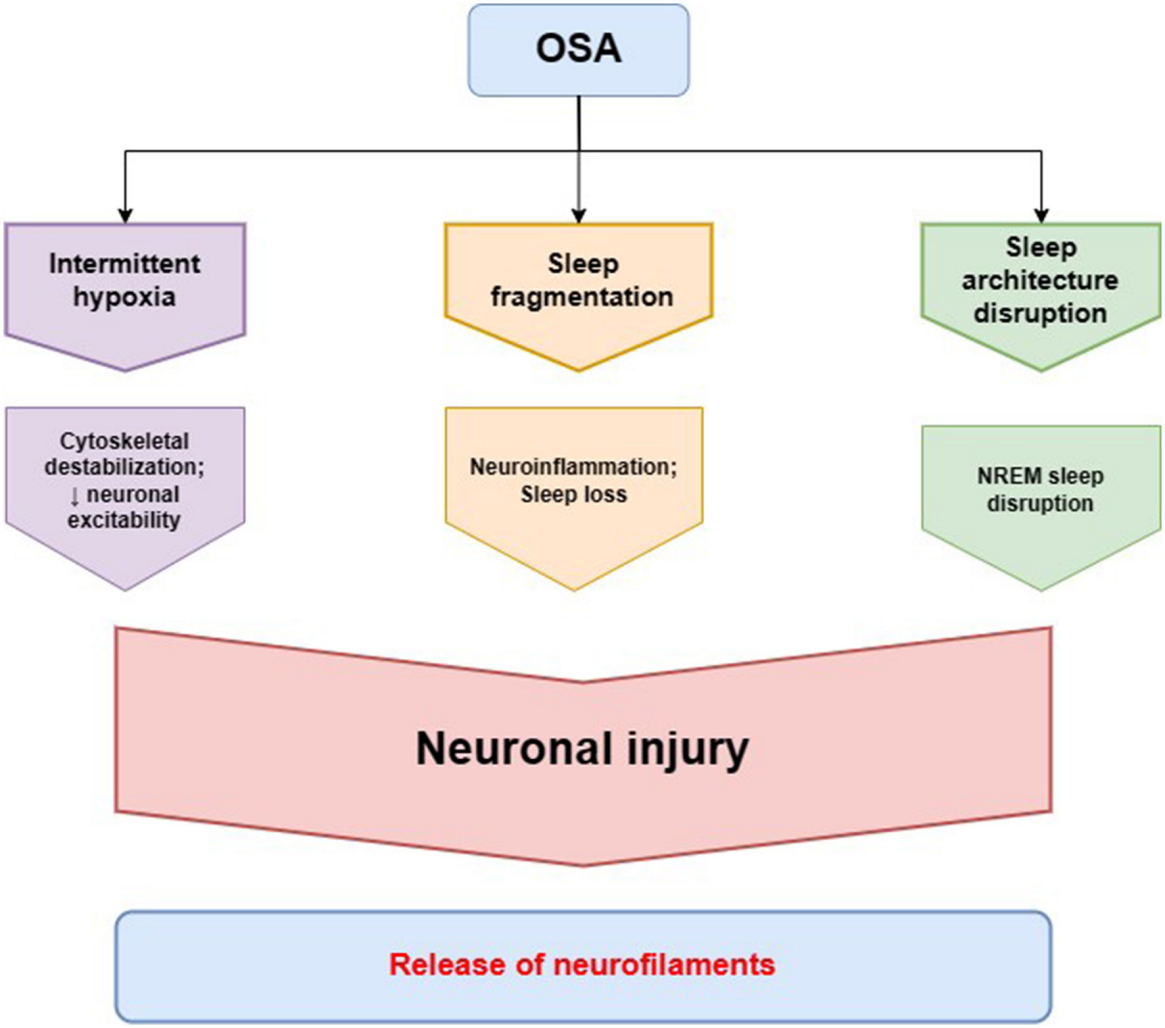
Pathomechanisms of obstructive sleep apnea (OSA) leading to alterations in neurofilament homeostasis. Intermittent hypoxia in OSA is the main factor involved in neuronal cytoskeletal destabilization that leads to decreased neuronal excitability and neuronal injury. Sleep fragmentation in OSA contributes to neurodegeneration through neuroinflammation and loss of the neuroprotective function of healthy sleep. The characteristic disruption of sleep architecture in OSA patients, especially non-rapid eye movement (NREM) sleep disruption, has also been described to be involved in the process of neurodegeneration. The cytoskeletal changes in neurons and neuronal injury lead to a release of neurofilaments, as a main cytoskeletal protein providing neuronal structural stability, to cerebrospinal fluid and peripheral blood.

NREM sleep loss was reported to trigger an increase in CSF and plasma NfL, while REM deprivation alone did not produce the change (56). It can be due to the disruption of NREM synaptic renormalization and cellular maintenance derived from the remodeling of cortical plasticity within slow-wave NREM sleep (57). The loss of N3 sleep can cause accelerated brain aging, resulting in lower cortical and subcortical brain volumes as observed in MRI (58). In patients with mild to moderate Alzheimer's disease, CSF NfL levels exhibited a positive correlation with stage 1 NREM sleep length and a negative correlation with stage 3 NREM sleep length (59). Patients with mild traumatic brain injury who experience poorer sleep quality were at a greater risk of developing OSA, measured by STOP-BANG scores, compared to subjects with better sleep quality. Additionally, poor sleepers had both increased peripheral NfL levels and lower cognitive performance (60). As OSA patients are likely to exhibit NREM sleep disruption in accordance to the scheme—stage N1 elongation and N3 diminution with an enhancement of alpha and beta wave frequency correlated with AHI—it may entail increase in extracellular NF levels (61–63). This pattern does not provide an adequate amount of slow-wave activity and transition into the REM phase, whereas light sleep expands and can cause more nocturnal awakenings, which, therefore, explains the increased tendency to daytime sleepiness complaints (64). In general, NREM sleep improves memory consolidation, reconsolidation, neurocognitive functions, and toxic waste removal, which is why being devoid of an appropriate amount of it can be reflected in CSF/blood NFs levels (see Figure 2).

## 4 Influence of OSA on neurofilaments

OSA through its main features, including CIH, sleep fragmentation, abnormal sympathetic activation, and low-grade inflammation, independently affects brain cells leading to cell stress and damage; however, CIH is the foremost triggering factor (see Figure 1). The latest data suggest nocturnal hypoxia is more associated with an increase in beta-amyloid 42 in the CSF fluid and exacerbated brain glucose uptake than with just sleep fragmentation (65, 66). Another influence of OSA on the central nervous system can be through disruption in the serotonergic system, as impaired serotonin neuromodulation affects both brainstem cardiorespiratory circuits and neurocognitive networks apart from its role in ventilatory stimulation (67–71). CIH and nitric oxide have been reported as inhibiting factors of tryptophan hydroxylase 1, one of the enzymes involved in serotonin synthesis from L-tryptophan (68). Thus, a decrease in serotonin synthesis observed among OSA patients can affect NFs' biology, but the potential connection hasn't been described yet.

### 4.1 Influence of intermittent hypoxia on neurodegeneration

It was reported that OSA may trigger neuroaxonal injury, particularly in the hippocampus as a hypoxia-sensitive area, resulting in ~6% gray matter loss during T1 signal MRI scan compared to healthy individuals (72). In addition, a 7-fold increase of apoptosis in the CA1 hippocampal region and an 8-fold increase in the adjacent cortex were detected in mice brains after 2 days of CIH exposure. Although the levels gradually decreased in 14 days, they were still elevated compared to controls (73). Cognitive decline is significantly influenced by the impact of CIH on cellular and chemical neuronal homeostasis (15, 74). In the brain of the intermittent hypoxia neonatal mouse model, the transcription of all NF subunits declined as follows: 20%—NfL and NfM, 30%—NfH (75). The degree of phosphorylation decreased in NfM and NfH, pointing to developed synaptic disruption as the effect of CIH on the neuronal network. Obtained cytoskeletal destabilization has put the NFs at a risk of proteolysis. The decreased neuronal excitability and impaired impulse conduction had been detected up to several weeks after exposure to CIH (75). In adult rats, CIH application during sleep resulted in spatial memory impairment, but not sensorimotor disturbances. Neurogenesis in the hippocampus measured by newly generated neurons immunohistochemical detection double labeling 5-bromo-2′-deoxyuridine connected with NFs decreased after early exposure and increased after 14 days of CIH, pointing to the presence of adaptive changes contributing to subsequent partial cognitive function improvement; however, the overall observation lasted only 30 days and the presumptive development of compensatory mechanisms in response to CIH exposure was not reflected by cognitive impairment amelioration (76). As untreated OSA is an independent risk factor for cognitive decline, it is conceivable that newly generated neurons degenerate within a few weeks of long-term CIH exposure, not alleviating neuronal damage (see Figure 1). Among humans, there was not any study directly measuring the condition of neuroplastic change; however, as brain imaging or behavioral tasks show, OSA patients do not experience improvement in the long term despite compensatory mechanisms, which may, in most cases remain insufficient without implemented treatment (77).

### 4.2 Influence of sleep fragmentation on neurodegeneration

Sleep fragmentation has been reported as a factor directly contributing to not only subjective cognitive decline but also neurodegenerative disease development (78). Animal studies on mice established sleep fragmentation dysregulates endosome-autophagy-lysosome protein degradation pathways and induces neuroinflammation on neural networks. The mice subjected to chronic sleep fragmentation showed an increase in amyloid-beta aggregation in the prefrontal cortex and hippocampus compared to controls; in tissues, there was an enlargement of lysosomes and increase in Ras-related proteins Rab5/Rab7, indicating enhanced endosome activity. In addition, microglia activation reflected by IBA1/CD68 levels participated in changes similar to early-stage Alzheimer's disease development. The study group showed lower scores in cognitive testing and had increased anxiety (79). In addition, fluorodeoxyglucose positron emission tomography exposed impaired brain energy metabolism as an increase of glucose uptake in various parts of the limbic system: the hypothalamus, the hippocampus, and the amygdala. In the hippocampus, hyperphosphorylated tau clusters and gliosis indicating susceptibility to Alzheimer's disease development were detected. However, the differential gene expression pattern in the hippocampus did not correlate with the Alzheimer's disease model, normal aging model, and APOE4 mutation model, but correlated with the stress model (80). In the Alzheimer's disease mice model, sleep fragmentation aggravated the disease severity with an increase in hippocampal beta-amyloid and brain neuroinflammation. Interestingly, there was minimal difference in tau and phosphorylated tau levels in mice with developed Alzheimer's disease and concomitant sleep fragmentation compared to those without. That is why the influence of sleep disruption on tau protein may be important only in the initial development and early stages of neurodegenerative diseases (81).

### 4.3 Neurofilament as a biomarker of cognitive impairment in OSA

The evaluation of serum NfL applicability as a general biomarker of brain condition measured as cognitive performance in OSA subjects was investigated only in the children's group. A retrospective study was conducted, wherein the group with both adenotonsillar hypertrophy and OSA exhibited higher NfL levels (31.68 pg/ml) compared to a group with adenotonsillar hypertrophy but without OSA (19.3 pg/ml) (82). The diagnostic discriminatory ability of the biomarker within the context of OSA was assessed as good with the area under the curve of 0.816 and with the use of a cutoff value of NfL of 18.75 pg/ml, resulting in 89.3% sensitivity and 61.2% specificity. NfL levels correlated with polysomnography parameters including Apnea-Hypopnea Index (AHI), Respiratory Arousal Index, and Oxygen Saturation Index, indicating a dependence between biomarker and OSA severity parameters. Cognitive status established using the Wechsler Intelligence Scale for Children (verbal, performance, and full-scale intelligence quotient measured) showed a significant correlation between serum levels of NfL and OSA-associated cognitive deficits (see Figure 2). In addition, intelligence parameters were associated with Oxygen Saturation Index and Respiratory Arousal Index, pointing to the fact that nocturnal severe hypoxemia and sleep fragmentation might be involved in substantial cognitive morbidity observed in OSA more than the number of apneas/hypopneas. Serum brain-derived tau protein as a neurodegenerative biomarker was measured as well; however, there was no significant difference between the group with and without OSA. Among adult OSA patients, NfL levels were correlated with the Oxygen Desaturation Index, but not with the AHI, indicating increasing hypoxia as a trigger of neuroaxonal damage; however, no neurocognitive assessment were performed (83). In another study, NfL levels of cognitively normal individuals with OSA did not correlate with the severity of the disease defined by the AHI values; it should be mentioned that the mean age of participants was 68.1, which makes the study not reliable in light of the current information that individuals with over 60 years are considered as having unstable biomarker levels due to occurring physiological aging (84, 85). Another research of cognitively normal middle-aged (median age 60.4 years) untreated OSA patients exhibited elevated NfL levels, associated with greater white matter hyperintensities quantified by magnetic resonance imaging and lower oxygen saturation—the AHI value was not related to NfL (86).

## 5 Effects of CPAP treatment on cognitive disorders in OSA patients

Research suggests that CPAP therapy among OSA patients can improve cognitive performance and have positive effects on both cardiovascular and metabolic risks (87–89). These changes can occur after reducing the occurrence of apneas/hypopneas and normalizing the sleep structure through sleep-dependent synaptic plasticity observed as brain volume increase (90). Brain imaging studies showed metabolite concentration markers change positively in response to CPAP treatment in mice, but not in humans (91, 92). The positive effect on the brain activity reflected by the task-positive network and default mode network was noted after 2 months of CPAP treatment among OSA patients (93). It is difficult to determine the level of reversibility and stability as there is a paucity of studies comprehensively focusing on brain plasticity and cognitive condition in response to CPAP in the short and long term. As observed, just one night without CPAP resulted in a decline of cognitive performance (94). It was observed whether CPAP therapy can impact serum levels of NfL, and the overnight change in biomarker levels was noted, whereas CPAP withdrawal resulted in an NfL increase, indicating the importance of early detection and institution of treatment (95).

## 6 Discussion

As described above, the indication of peripheral NF levels among OSA patients can support traditional cognitive performance testing, extending the scope of knowledge about a patient's neuropsychological condition. Neurofilament biology and measurement of the ease of levels predispose them to be examined for utility as cognitive biomarkers. Although plenty of potential biomarkers have been tested so far (with the most significant including amyloid-β 42, phosphorylated tau, and total tau), they are not precise and thereby not clinically useful. What is important is that NFs directly reflect the current neural condition as they are part of the neuronal structure. They can easily diffuse through the blood-brain barrier to peripheral blood, which makes them easy and cheap to determine reliable biomarkers. Despite many hypothetical profits, the method has its limitations. First, there is only one study comparing the NF levels and cognitive performance among OSA patients; thus, in the case of this disease, the topic is not explored enough (see Table 1). Additionally, the study was carried out on children, not on adult OSA samples. Other studies comparing NF levels among OSA patients focused more on general dependencies between NF levels and polysomnographic features or the response to CPAP treatment. There was not any research comparing both cognitive performance and NF levels before and after CPAP application, but there were only indirect connections between them. Second, the indication of serum NF levels requires taking a blood sample and laboratory testing. Although the levels of NFs measured in peripheral blood are close to those in the cerebrospinal fluid, the differences can impact reasoning and comparing levels in cognitive change. In addition, the assessment of NF levels should not be the only method used in the cognition measurement; the biomarker can be the most useful while checking the progress of the disease and the influence of the instituted treatment. Therefore, the concept of using NFs as cognitive biomarkers should support traditional cognitive testing, providing a more precise description of the patient's cognitive performance.

**Table 1:** Studies concerning serum neurofilament light (NFL) levels in obstructive sleep apnea (OSA).

**References**	**Study group**	**Control group**	**Study group mean and standard deviation NFL level (pg/ml)**	**Control group mean and standard deviation NFL level (pg/ml)**	***P*-value**
Michael et al. (85)	56 children with OSA	49 children without OSA	31.68 (27.29–36.07)	19.13 (17.32–20.95)	<0.001
Kam et al. (95)	First group: 26 adults with mild OSA, second group: 32 adults with moderate-severe OSA	23 adults without OSA	First group: 95.29 (77.27–113.31), second group: 93.81 (75.91–111.71)	92.29 (73.79–110.79)	0.847
Sprecher et al. (86)	Six adults with moderate-severe OSA		22 adults without OSA			Exact data not given; performed *t*-test showed increased NFL levels in the study group (*t* = 2, 2)		0.04
Arslan et al. (83)	30 adults with OSA before and after 3-night CPAP withdrawal		–			Exact data not given; noticed NFL increase after CPAP withdrawal	–		<0.05
Shi et al. (82)	First group: 20 adults with mild OSA, second group 11 adults with moderate OSA, third group: 10 adults with severe OSA	29 adults without OSA		Exact data not given		0.67			

## 7 Conclusions

OSA and pathological changes associated with it may contribute to the neurodegenerative processes, making it an independent risk factor for cognitive decline. It has been previously described that both CIH and sleep fragmentation have a significant impact on neuronal homeostasis and cognition. CIH is thought to be responsible for neuroaxonal injury and apoptosis in hippocampal regions responsible for cognitive functioning through cytoskeletal destabilization. On the other hand, sleep fragmentation may induce neuroinflammation leading to neurodegeneration and cognition impairment. Both processes could possibly trigger the increase in the concentration of neurospecific biomarkers of neurodegeneration, such as NFs in plasma and CSF, which could serve as a predictor of cognitive decline. The recent studies on the correlation of NFs and OSA show that NF plasma concentration is positively correlated with the polysomnographic parameters of OSA severity, although there is only one study in the pediatric population showing the dependence of NF concentration and the significant decline in cognition in OSA patients. The emerging data suggest that CPAP therapy may also influence the NF concentration and lead to the improvement of cognitive functions in OSA patients. To our knowledge, this study is the first review to focus on the role of NFs in the neurodegenerative processes in OSA and its possible role as a biomarker of cognitive decline in OSA patients, comprehensively describing most recent studies. In light of these observations, it is important to further investigate NFs as the possible early biomarkers of neurodegeneration and cognitive impairment in OSA patients to properly monitor, adjust, and implement accurate therapy and its efficiency. From a future perspective, exploring the neurofilaments could provide an objective assessment of cognitive function decline; however, more studies are needed to be performed. New directions of research include comparisons between cognitive testing tools and NF levels, measured both in peripheral blood and CSF in OSA patients before and after CPAP treatment. Additionally, the disruption of NF gene expression might play a role in cognitive impairment development, which needs to be examined.

## Author contributions

JJ: Conceptualization, Investigation, Writing – original draft. PK: Investigation, Writing – original draft, Visualization. DS: Writing – review & editing. MS: Writing – review & editing. PB: Writing – review & editing. AG: Conceptualization, Writing – review & editing.
